# The New York Medical School

**Published:** 1850-08

**Authors:** 


### THE NEW YORK MEDICAL SCHOOL.

Recent advices from the city of New York inform us that Dr. Mott transmitted his resignation of the Chair of Surgery in the New York Medical School, from Europe, upon hearing of the election of Dr. Detmold to the Chair of Theory and Practice. This was immediately followed by the resignation of Dr. Detmold, and thus the two most important Chairs of the Medical Department of the University of New York are withont occupants, and the school is almost in a state of chaos.

It is a pity these revolutions did not take place before the circular of the New York school was published, because it is troublesome to get up new machinery for the attraction of students. The apparatus of a surgical clinique every Saturday, by Professor Mott, and a medical and surgical clinique every Wednesday by Professors Patterson and Detmold is completely disarranged by the pettish resignations announced above. Who is to take charge of these fractured cases it is hard to say, and we suppose the trustees will find some difficulty in determining who will do so. B.

